# *microRNA-135a-5p* regulates *NOD-like receptor family pyrin domain containing 3* inflammasome-mediated hypertensive cardiac inflammation and fibrosis *via thioredoxin-interacting protein*

**DOI:** 10.1080/21655979.2021.2024956

**Published:** 2022-02-11

**Authors:** Hao Chen, Huilian Qiao, Qiang Zhao, Fuling Wei

**Affiliations:** a Department of Cardiovascular Center, The 8th Medical Center of General Hospital of PLA, Beijing, China; b Department of Pathology, Air Force Medical Center PLA, Beijing, China

**Keywords:** Hypertension, cardiac fibrosis, *miR-135a-5p*, *TXNIP*, *NLRP3*, cardiac inflammation, proinflammatory factor, protein-protein interaction

## Abstract

Hypertension is a severe public health problem that induces cardiac injury with alterations of gene expressions. The current study sought to evaluate the mechanism of *microRNA*(*miR)-135a-5p* in NOD-like receptor family pyrin domain containing 3 (*NLRP3*) inflammasome-mediation of cardiac inflammation and hypertensive cardiac fibrosis. Firstly, hypertensive mouse models were established using angiotensin II (Ang II), followed by *miR-135a-5p* agomir treatment. Subsequently, mouse blood pressure and basic cardiac function indexes, histopathological changes, and cardiac fibrosis were all determined, in addition to detection of factors related to inflammation and fibrosis. Additionally, mice cardiac fibroblasts (CFs) were isolated and treated with Ang II. The binding relationship of *miR-135a-5p* and thioredoxin-interacting protein (*TXNIP*) was predicted and testified, while the interaction of *TXNIP* and *NLRP3* was detected by means of a co-immunoprecipitation assay. It was found that *miR-135a-5p* was poorly-expressed in Ang II-treated mice and further exerted cardioprotective effects against hypertensive heart diseases. Moreover, over-expression of *miR-135a-5p* resulted in inhibition of inflammatory infiltration and almost eliminated cardiac fibrosis, as evidenced by decreased Collagen (COL)-I, COL-III, a-smooth muscle actin, *NLRP3*, tumor necrosis factor-α, and interleukin-6. Mechanically, *miR-135a-5p* inhibited *TXNIP* expression to block the binding of *TXNIP* and *NLRP3*. On the other hand, *TXNIP* up-regulation reversed the protective role of *miR-135a-5p* over-expression in CFs. Collectively, our findings indicated that *miR-135a-5p* over-expression inhibited *TXNIP* expression to block the binding of *TXNIP* and *NLRP3*, thereby alleviating hypertensive cardiac inflammation and fibrosis.

## GRAPHICAL ABSTRACT

**Figure:**
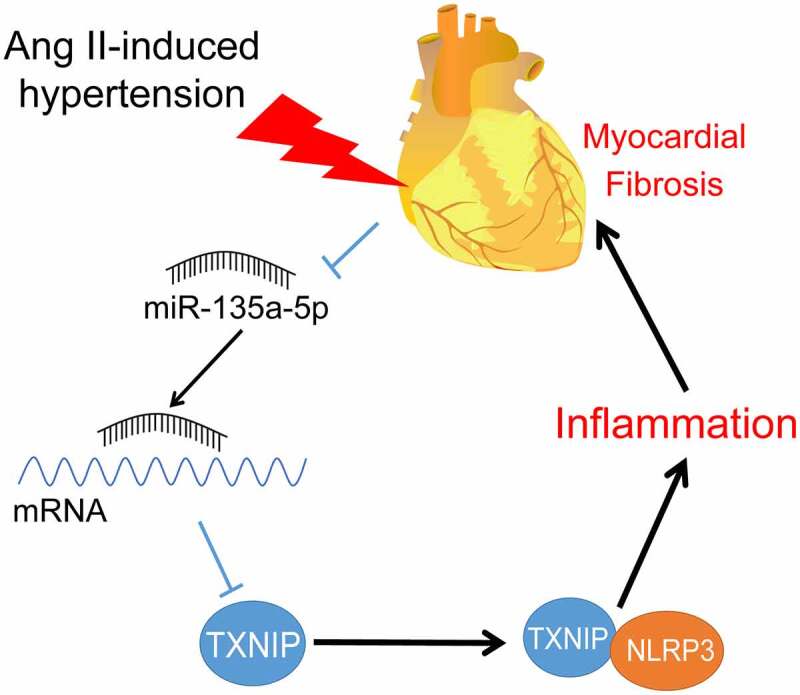

## Introduction

Hypertension poses a major risk factor for a myriad of heart diseases, including left-sided heart disease and valvular heart disease, ultimately leading to heart failure and decreased quality of life [1–3]. Increased blood pressure brings about enhanced muscle mass of the left ventricle (LV) and promotes extracellular matrix (ECM) remodeling and tissue stiffness, consequently precipitating cardiac fibrosis [4]. Inherently, cardiac fibrosis is defined as the collagenous scar tissue produced by the deposition of extracellular matrix proteins, and further serves as a common pathological process of cardiac diseases [5,6]. Besides, hypertension can also trigger numerous chronic inflammatory responses, contributing to the exacerbation of cardiac fibrosis [7]. Meanwhile, there is a lack of effective therapeutic targets in the treatment of cardiac fibrosis due to dose limit and toxicity [8]. In lieu of the same, it would be prudent to address the alleviation of hypertension-induced cardiac inflammation and fibrosis in the form of the discovery of novel targets.

NOD-like receptor family pyrin domain containing 3 (*NLRP3*) represents a multiprotein oligomer primarily enriched in activated immune and inflammatory cells [9]. Interestingly, the *NLRP3* inflammasome is capable of mediating the production of apoptosis and inflammation-related proteins to participate in hypertension-induced end-organ damage [10,11] Moreover, *NLRP3* is attributed to the inflammatory response in fibrosis of numerous organs, including the liver, kidney, lung, and heart [12–15]. More importantly, there is evidence to suggest that the *NLRP3* inflammasome contributes to tissue scarring and cardiovascular healing *via* overproduction of interleukin (IL)-1β and IL-18 during cardiac fibrosis [12]. Therefore, inhibition of *NLRP3* inflammasome could prove a potentially effectivestrategy for the treatment of hypertensive cardiac fibrosis.

MicroRNAs (miRNAs), a type of short RNAs with 18 to 25 nucleotides, are involved in the alteration of cardiac functions, such as *miR-145-5p, miR-337-5p, miR-122-5p* [16–18]. One such miRNA, namely miR-135a, is known to play a protective role in the life-cycle of cardiomyocytes and exerts cardioprotective both in cardiac injury and ischemia-reperfusion injury [19–21]. What’s more, prior studies have detected down-regulation of miR-135a upon isoproterenol-induced cardiac fibrosis, and further suggested miR-135a confer a therapeutic role in cardiac fibrosis treatment [22,23]. Interestingly, *miR-135a-5p* possesses the ability to negatively modulate the expression of *NLRP3 via* suppression of thioredoxin-interacting protein (*TXNIP*) [24]. *TXNIP*, an intracellular inhibitor of the thioredoxin system, is known to participate in the impairment of glucose homeostasis and the etiology of cardiovascular dysfunction, including hypertension [25]. Meanwhile, the inactivation of *TXNIP* also exerts a restrictive effect on angiotensin II (Ang II)-induced cardiac fibrosis and hypertrophy [26]. Nevertheless, the crosstalk of *miR-135a-5p* and *NLRP3* inflammasome/*TXNIP* in cardiac fibrosis has not been discussed before and requires further exploration.

Based on the aforementioned data and findings, we hypothesize that *miR-135a-5p* might regulate *NLRP3* inflammasome-mediated cardiac inflammation to alleviate cardiac fibrosis *via* modulation of the *TXNIP* expression. In order to validate our hypothesis, we established hypertensive mouse and cell models by means of Ang II treatment to explore the role of *miR-135a-5p* and its downstream mechanism in AD. In this manner, the current study set out to analyze the functional mechanism of *miR-135a-5p* in cardiac inflammation, in an effort to provide a novel theoretical basis for cardiac fibrosis treatment.

## Methods and material

### Animal grouping

A total of 24 C57BL/6 male mice (aged 8 weeks) (HUAFUKANG Bioscience Co., Beijing, China) were procured and raised in a constant temperature and humidity environment (temperature: 20 ± 1°C; humidity: 60 ± 10%; light cycle: 12 h), with *ad libitum* access to food and water. The mice were randomly allocated into the following 4 groups: the sham group, the Ang II group, the Ang II+agomir NC group, and the Ang II+*miR-135a-5p* agomir group. All animal experimentation in the current study conformed to the Guidelines for The Care and Use of Laboratory Animals published by the National Institutes of Health (NIH) [27], and was approved by the Animal Ethics Committee of the 8th Medical Center of General Hospital of PLA. Extensive efforts were made to minimize the number and suffering of the included animals.

### *Treatment of Ang II and intramyocardial injection of* miR-135a-5p *agomir*

To induce hypertension, the C57BL/6 J male mice were infused with Ang II using ALZET osmotic pumps (model 1007D; Cupertino, CA, USA) (at a dosage of 1.5 mg/kg/day) as described in previous research [28]. Prior to Ang II treatment, the mouse chest was dissected on the fourth rib. Subsequently, the mice were intramyocardially injected with agomir NC and *miR-135a-5p* agomir (50 μL, 300 nmol/kg) (Guangzhou RiboBio Co., Ltd, Guangzhou, Guangdong China) [29]. Simultaneously, mice in the sham group were treated with the same amounts of normal saline as the control.

### Blood pressure and cardiac functions

Blood pressure and cardiac function indices were assessed after 2 weeks of Ang II treatment. Blood pressure readings [systolic blood pressure (SBP), diastolic blood pressure (DBP)] were obtained using a noninvasive tail-cuff blood pressure system (Muromachi Kikai Co, Ltd, MK-2000ST NP-NIBP Monitor, Tokyo, Japan). Mean arterial pressure (MAP) was calculated using the following formula: MAP = (SBP+2× DBP)/3 [30].

Thereafter, the mice were lightly anesthetized with an intravenous injection of ketamine (50 mg/kg) and xylazine (50 mg/kg). Subsequently, LV functions were assessed by means of two-dimensional echocardiography using a Vevo770 high-resolution ultrasound imaging system (VisualSonics, Toronto, ON, Canada) equipped with an RMV 707B scan head (30 MHz) (VisualSonics). Briefly, the mice were examined on the left lateral position in the anterior chest wall and LV functions were analyzed using parasternal long- and short- axes. Cardiac functions of mice (body temperature: at 37°C, heart rate: 450 times/min) were reflected by detection results of left ventricular ejection fraction (LVEF), left ventricular fraction shortening (LVFS), and LV mass [31] based on the manufacturer’s guidelines of Vevo 770 system. Afterward, the mice were sacrificed with an intraperitoneal injection of ketamine (1 g/kg) and xylazine (100 mg/kg) to obtain cardiac tissues for subsequent biochemical and histopathological analyses [28].

### Histological staining

Referring to prior research [32], following fixation with 4% paraformaldehyde and paraffin-embedding, the cardiac tissues were sliced into sections (thickness of 5 μm) and cultured with hematoxylin and eosin solution (H&E, Sigma, St Louis, MO, USA) and Masson’s trichrome (Polysciences, Bay Shore, N.Y., USA) for observation of the myocardial histopathological structure and myocardial fibrosis.

All cardiac sections were collected and stored digitally using a Nikon slide scanner. To evaluate myocardial fibrosis, 20X Massons-Trichrome images of the entire LV were exported, and the Image-J software (National Institutes of Health, Bethesda, MD, USA) was adopted to calculate the collagen fraction (%) as the ratio of the total fibrosis area (blue) to the total tissue area. The above-mentioned experiments were performed by independent technicians who were blinded to mice grouping.

### Enzyme-linked immunosorbent assay (ELISA)

Referring to prior research [33], the cardiac tissues were ground on ice to prepare a homogenate, followed by centrifugation at 10,000 g for 10 min to obtain the supernatant. Subsequently, cardiac fibroblasts (CFs) from all groups were harvested and rinsed with phosphate buffer saline (PBS) 3 times. After centrifugation at 1200 g for 5 min, the CFs were cultured with PBS containing cell lysates at 4°C for 10 min, followed by another round of centrifugation at 170,000 g for 5 min to obtain the supernatant. Afterward, the contents of inflammatory factors [tumor necrosis factor (TNF)-α and IL-6], Collagen (COL)-I, and COL-III were measured with the help of ELISA kits (Cusabio Biotech, Newark, USA).

### Isolation and treatment of mouse cardiac fibroblasts (CFs)

CFs were isolated from the LV of C57/BL6 mice and characterized with positive expression levels of vimentin and negative expression levels of desmin (a smooth muscle marker) and factor VIII (an endothelial cell marker) [34,35]. CFs (N = 1 × 10^7^) of the third generation were used for subsequent experimentation [31]. Next, the CFs were subjected to transfection with *miR-135a-5p* mimic (50 pmol), *TXNIP* over-expression plasmid (0.2 μM, oe-*TXNIP*), and corresponding controls (GenePharma, Shanghai, China) using the Lipofectamine 2000 reagent (Invitrogen, Carlsbad, CA, USA). After a 24 h [36] period of transfection, the cells were treated with 100 nM AngII for 24 h before subsequent analyses.

### Reverse transcription quantitative polymerase chain reaction (qRT-PCR)

Referring to prior research [37], total RNA content was extracted from the cells (N = 1 × 10^7^) and tissues using RNeasy Mini kits (Qiagen, Valencia, CA, USA), and then reverse-transcribed into the complementary DNA (cDNA) with reverse transcription kits (RR047A, Takara, Tokyo, Japan). To detect miRNA, the miRNA was reverse-transcribed into cDNA using miRNA First Strand cDNA Synthesis (Tailing Reaction) kits (B532451-0020, Sangon Biotech, Shanghai, China). Subsequently, qRT-PCR was carried out using YBR® Premix Ex Taq^TM^ II (Perfect Real Time) kits (DRR081, Takara) and real-time fluorescence quantitative PCR apparatus (ABI 7500, ABI, Foster City, CA, USA). PCR was amplified with the following two steps: the first step was pre-denaturation at 95°C for 30 s, and the second step was PCR under 40 thermal cycles (95°C for 5 s and 60°C for 34 s). Each sample was set with 3 duplicate wells. qPCR primers were provided by Sangon Biotech (Table 1). Ct value of each well was recorded with GAPDH or U6 serving as the internal reference. The relative gene expression was calculated using the 2^−ΔΔCt^ method [38]. ΔΔCt = (mean Ct value of target genes from the experiment group – mean Ct value of housekeeping genes from the experiment group) – (mean Ct value of target genes from the control group – mean Ct value from housekeeping genes of the control group).

**Table 1.** qPCR primers

Gene	Forward Primer (5'-3')	Reverse Primer (5'-3')
miR-135a-5p	AACCCTGCTCGCAGTATTTGAG	GCGGCAGTATGGCTTTTTATTCC
TXNIP	AGTGATTGGCAGCAGGTC	GGTATCTGGGATGTTTAGG
IL-6	TAGTCCTTCCTACCCCAATTTC	TTGGTCCTTAGCCACTCCTTC
TNF-α	CCTCCCTCTCATCAGTTCTA	ACTTGGTGGTTTGCTACGAC
U6	CTCGCTTCGGCAGCACA	AACGCTTCACGAATTTGCGT
GAPDH	CAGTCACTACTCAGCTGCCA	GAGGGTGCTCC GGTAG

### Western blot assay

Referring to prior research [37], tissues (5 mg) or cells (1 × 10^7^) were lysed with an enhanced RIPA lysis solution containing protease inhibitor (Boster Biological Technology Co. Ltd, Wuhan, China). Next, the protein concentration was quantified using bicinchoninic acid (BCA) protein quantification kits (Boster Biological Technology Co. Ltd). Subsequently, the proteins were isolated using 10% sodium dodecyl sulfate polyacrylamide gel electrophoresis (SDS-PAGE) and electrically-transferred onto polyvinylidene fluoride (PVDF) membranes. Afterward, the membranes were blocked with 5% bovine serum albumin at room temperature for 2 h to block the nonspecific binding, followed by incubation with diluted primary anti-rabbit *TXNIP* (# 14715S, Cell Signaling Technology, Danvers, MA, USA), *NLRP3* (ab263899, dilution ratio of 1:1000), and β-actin (ab8227, dilution ratio of 1:2000) respectively at 4°C overnight. Following washing, the membranes were cultured with the secondary horseradish peroxidase-labeled goat anti-rabbit IgG (ab205718, dilution ratio of 1:2000) at room temperature for 1 h. With the exception of *TXNIP*, all the aforementioned antibodies were provided by Abcam (Cambridge, MA, USA). The Western blots were visualized following the addition of an enhanced chemiluminescence working solution (EMD Millipore, Billerica, MA, USA). The gray value of each band in Western blot images was quantified using the Image Pro Plus 6.0 software (Media Cybernetics, San Diego, CA, USA), with β-actin serving as the internal reference. Experiments in each group were performed 3 times to obtain the mean value.

### Immunofluorescence

Referring to prior research [30], separated cells were subjected to immunofluorescent staining according to the provided instructions. To detect α-SMA expression pattern in CFs, cells of each group were seeded into coverslips containing the medium and cultured in a humidified incubator with 5% CO_2_ and 95% air at 37°C. After the slides were covered by cells, the cells fixed in 4% paraformaldehyde were incubated with anti-rabbit α-SMA (ab124964, dilution ratio of 1:250, Abcam) in a humid and dark room at 4°C. Subsequently, the coverslips were washed with 0.1% Triton-X and 1× PBS three times with 5 min of each time, and then incubated with goat anti-mouse IgG (Alexa Fluor® 488) (ab150077, dilution ratio of 1: 500, Abcam) in a humid and dark room at 4°C for 1 h. Afterward, fluorescence images were obtained using a fluorescence microscope. The fluorescence intensity of α-SMA was quantified using the Image-Pro Plus 6.0 software and expressed as the relative value.

### Dual-luciferase reporter assay

The binding sites of *miR-135a-5p* and *TXNIP* were predicted with the help of the ENCORI: The Encyclopedia of RNA Interactomes. (sysu.edu.cn) website [39]. Next, the *TXNIP* 3ʹUTR fragments containing the binding sites of *miR-135a-5p* (*TXNIP*-WT) and *TXNIP* 3ʹUTR containing mutated binding sites of *miR-135a-5p* (*TXNIP*-WT) were cloned into the pMIR-reporter plasmids (Beijing Huayueyang Biotechnology, Beijing, China). Subsequently, constructed luciferase reporter plasmids and mimic NC or *miR-135a-5p* mimic were co-transfected into mouse CFs (Shanghai Beinuo Biotechnology, Shanghai, China). After 48 h, cells lysates were prepared, followed by detection of luciferase activity using assay kits (K801-200, Biovision, Mountain View, CA, USA).

### RNA-binding protein immunoprecipitation (RIP)

Referring to prior research [37], the binding of *miR-135a-5p* and *TXNIP* was detected using RIP assay kits. Next, cells (N = 1 × 10^7^) from each group were rinsed with pre-cooled PBS and the supernatant was discarded. Subsequently, the cells were ice-bathed with an equal volume of RIPA lysis solution (P0013B, Beyotime, Shanghai, China) before centrifugation at 4°C for 10 min to obtain the supernatant. A portion of cell extracts was used as the Input and the remaining were co-precipitated with antibodies. Following sample digestion with protease K, RNA content was extracted for subsequent detection of *miR-135a-5p* and *TXNIP*. AGO2 antibody (ab32381, dilution ratio of 1: 100, Abcam) were mixed for 30 min at room temperature with anti-rabbit IgG (ab109489, dilution ratio of 1: 100, Abcam) as the negative control. Experiments in each group were performed 3 times to obtain the mean value.

### Co-immunoprecipitation (Co-IP)

Referring to prior research [40], cells (N = 1 × 10^7^) from each group were lysed with a lysis buffer (50 mM Tris-HCl (pH 7.4), 150 mM NaCl, 10% glycerinum, 1 mM ethylene damine tetraacetic acid, 0.5% NP – 40 and protease inhibitor), and the cell debris was removed by means of centrifugation. After the concentration of lysis solution was determined with a BCA reagent, equal amounts of protein were collected from each group and supplemented with the same volume of cell lysis solution. Subsequently, each sample was incubated with the following antibodies: *TXNIP* (# 14715S, dilution ratio of 1:50, Cell Signaling Technology), *NLRP3* (ab263899, dilution ratio of 1: 30, Abcam), and 15 μL protein A/G beads (Santa Cruz Biotechnology, Santa Cruz, CA, USA) for 2 h. After 3 rounds of washing, the beads were harvested using centrifugation, added with an equal volume of reducing loading buffer, and boiled at 100°C for 5 min. Afterward, the samples were isolated with SDS-PAGE and transferred onto PVDF membranes (Millipore, Temecula, CA, USA) for Western blot analysis.

### Statistical analysis

SPSS21.0 statistical software (IBM Corp, Armonk, NY, USA) and GraphPad Prism 9.0 software (GraphPad Software Inc., San Diego, CA, USA) were adopted for data statistical analyses and graphing, respectively. Measurement data were expressed as mean ± standard deviation (SD), and in line with normal distribution and homogeneity of variance. The *t* test was adopted for pairwise comparisons, while one-way or two-way analysis of variance (ANOVA) was utilized for multi-group comparisons, and Tukey’s multiple comparisons test was adopted for posttest checking. *P* value was obtained from two-sided tests. A value of *P* < 0.05 was regarded statistically significant.

## Results

The current study set out with the establishment of hypertensive mouse and *in vitro* cell models using treatment of Ang II, and subsequent experimentation revealed that *miR-135a-5p* expression levels were decreased in tissues and cells upon modeling, whereas *miR-135a-5p* over-expression alleviated cardiac fibrosis and inflammation in hypertensive mice. Mechanically, *miR-135a-5p* inhibited *TXNIP* expression to reduce the binding of *TXNIP* to *NLRP3*, thereby suppressing cardiac fibrosis and inflammation.

### miR-135a-5p *over-expression alleviated cardiac fibrosis of hypertensive mice*

**Figure 1.**
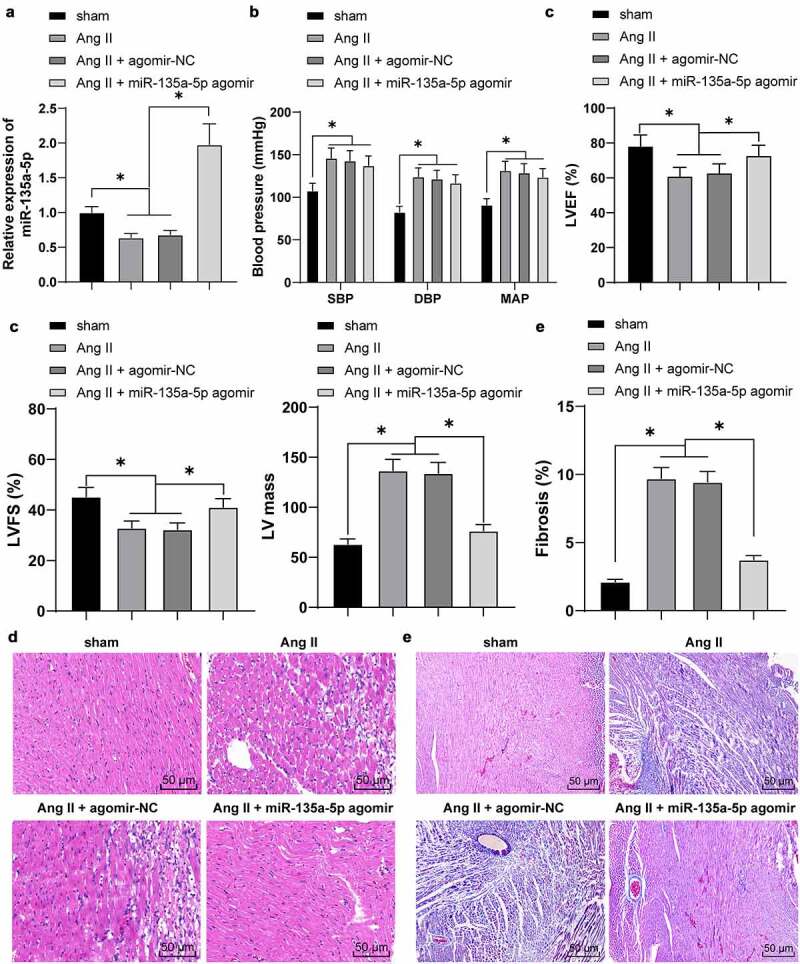
*miR-135a-5p* overexpression alleviated cardiac fibrosis of hypertensive mice. At 24 h before establishment of the hypertensive mouse model, mice were intramyocardially injected with *miR-135a-5p* agomir. (a): *miR-135a-5p* expression detected via RT-qPCR; (b): Blood pressure, including systolic blood pressure (SBP), diastolic blood pressure (DBP) and mean arterial pressure (MAP); (c): Basic cardiac function indexes; (d): Histopathological changes of cardiac tissues observed via H&E staining; (e): Cardiac fibrosis observed via Masson staining. N = 6, data were represented as mean ± SD. Data in figures A, C and E were analyzed using one-way ANOVA and data in figure B were analyzed using two-way ANOVA, followed by Tukey’s multiple comparison test. * P < 0.05.

Existing evidence suggests that miR-135a is capable of alleviating cardiac fibrosis [20]. However, its regulatory mechanism on hypertensive cardiac fibrosis remains elusive. Following the establishment of hypertensive mouse models, *miR-135a-5p* expression patterns were detected by means of RT-qPCR. It was found that *miR-135a-5p* expression levels were markedly reduced in cardiac tissues of Ang II–induced hypertensive mice (*P* < 0.05, Figure 1(a)).

To further explore the function of *miR-135a-5p* on cardiac fibrosis, the hypertensive mice were intramyocardially injected with *miR-135a-5p* agomir to over-express *miR-135a-5p* (*P* < 0.05, Figure 1(a)). Subsequently, blood pressure and basic cardiac function indexes were detected, which revealed that Ang II markedly increased blood pressure (*P* < 0.05, Figure 1(b)), accompanied by reduction in LVEF and LVFS and an increase in LV mass (*P* < 0.05, Figure 1(c)), whereas *miR-135a-5p* agomir reversed the aforementioned effects of Ang II on inhibiting LVEF and LVFS and promoting LV mass (*P* < 0.05, Figure 1(c)). Together, these findings suggested that *miR-135a-5p* agomir exerts cardioprotective functions in established hypertensive heart disease.

To further elucidate the role of *miR-135a-5p* agomir in cardiac functions of Ang II–induced hypertensive mice, we detected the histopathological changes of cardiac tissues in much detail. The results of H&E staining illustrated that compared with the sham group, the cells were disorderly arranged, cardiac fibers were obviously fracted, in addition to increased infiltration of pro-inflammatory cells in Ang II-treated mice. Following treatment of *miR-135a-5p* agomir, there was a marked reduction in Ang II–induced inflammatory infiltration (Figure 1(d)). Meanwhile, Masson staining illustrated that Ang II markedly facilitated cardiac fibrosis, whereas *miR-135a-5p* agomir almost eliminated the cardiac fibrosis (*P* < 0.05, Figure 1(e)). Collectively, these findings indicated that *miR-135a-5p* could alleviate cardiac fibrosis of hypertensive mice and exert cardioprotective functions.

### miR-135a-5p *over-expression attenuated cardiac inflammation of hypertensive mice*

**Figure 2.**
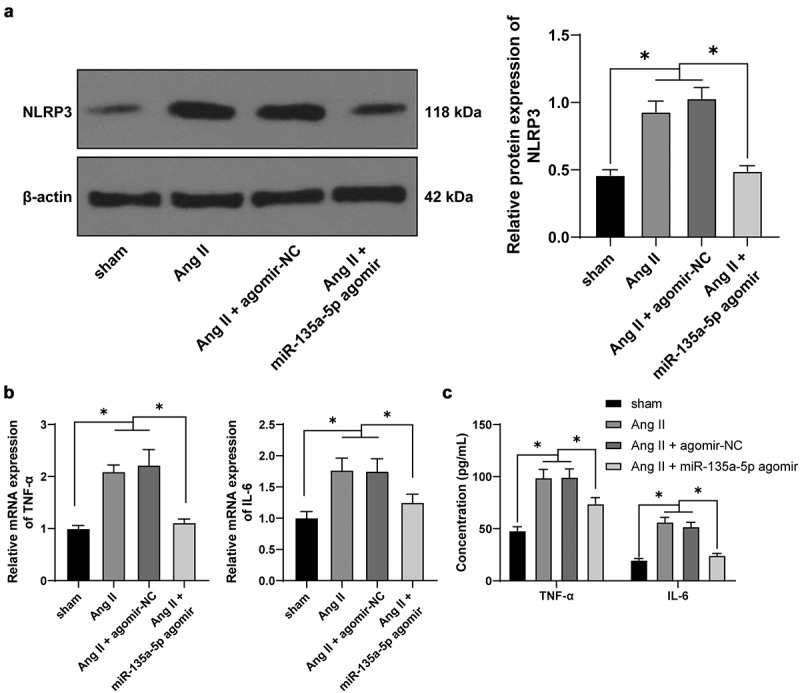
*miR-135a-5p* overexpression attenuated cardiac inflammation of hypertensive mice. a: *NLRP3* expression in cardiac tissues detected via Western blot analysis; b-c: TNF-α and IL-6 levels in cardiac tissues determined via RT-qPCR and ELISA. N = 6, data were represented as mean ± SD. Data in figure A were analyzed using one-way ANOVA and data in figures b-c were analyzed using two-way ANOVA, followed by Tukey’s multiple comparison test. * P < 0.05.

As the process of inflammation confers a significantly stimulating role in hypertensive cardiac remodeling, blocking the activation of *NLRP3* inflammasome could relieve cardiac inflammation and fibrosis [41]. Herein, the results of Western blotting showed that Ang II significantly increased the *NLRP3* expression, whereas *miR-135a-5p* agomir exerted the opposite role (*P* < 0.05, Figure 2(a)). Meanwhile, RT-qPCR and ELISA results further illustrated that TNF-α and IL-6 levels were markedly augmented by Ang II while diminished upon *miR-135a-5p* agomir (*P* < 0.05, Figure 2(b,c)). Together, these findings indicated that *miR-135a-5p* over-expression inhibited the levels of *NLRP3* and inflammatory cytokines to relieve cardiac inflammation and fibrosis.

### miR-135a-5p *over-expression suppressed Ang II–induced inflammation and fibrosis of cardiac fibroblasts (CFs)*

**Figure 3.**
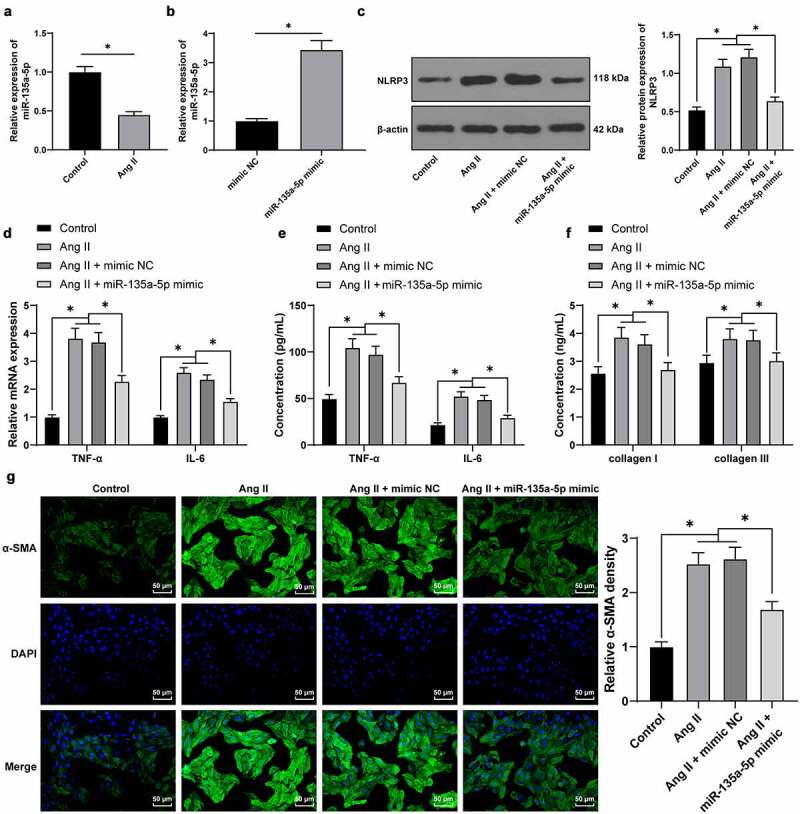
*miR-135a-5p* overexpression suppressed Ang II–induced fibrosis and inflammation of CFs. Mouse CFs were treated with Ang II and then transfected with *miR-135a-5p* mimic. a-b: *miR-135a-5p* expression detected via RT-qPCR; C: *NLRP3* expression determined via Western blot analysis; d-e: TNF-α and IL-6 levels measured via qRT-PCR and ELISA; f: COL-I and COL-III expression determined via ELISA; g: a-SMA level detected via immunofluorescence. Cell experiments were performed 3 times, and data were represented as mean ± SD. Data in figures a-b were analyzed using t test. Data in figures c and g were analyzed using one-way ANOVA. Data in figures B-C were analyzed using two-way ANOVA, followed by Tukey’s multiple comparison test. * P < 0.05.

To further validate the role of *miR-135a-5p* in hypertensive cardiac fibrosis, we isolated and cultured mouse CFs [31], which were subjected to treatment with Ang II. Subsequent RT-qPCR results illustrated that *miR-135a-5p* expression levels were markedly decreased as a result of Ang II treatment (*P* < 0.05, Figure 3(a)). Afterward, Ang II-treated CFs were transfected with *miR-135a-5p* mimic to over-express *miR-135a-5p* mimic (*P* < 0.05, Figure 3(b)). The results of Western blotting, RT-qPCR, and ELISA, all revealed that Ang II markedly facilitated the expressions of *NLRP3*, TNF-α, and IL-6, while *miR-135a-5p* mimic inhibited the promoting role of Ang II on the release of inflammatory factors (*P* < 0.05, Figure 3(c-e)), which highlighted the ability of *miR-135a-5p* to suppress Ang II–induced inflammation of CFs.

Furthermore, we adopted ELISA to detect the expression patterns of COL-I and COL-III, which revealed that Ang II promoted the expressions of COL-I and COL-III, while *miR-135a-5p* mimic reversed the aforementioned effects of Ang II (*P* < 0.05, Figure 3(f)). Moreover, immunofluorescence detection showed that Ang II elevated a-SMA levels, while *miR-135a-5p* mimic inhibited the encouraging role of Ang II on a-SMA (*P* < 0.05, Figure 3(g)). Together, the above findings indicated that *miR-135a-5p* suppressed Ang II–induced inflammation and alleviated fibrosis of CFs.

### miR-135a-5p *negatively-regulated* TXNIP

**Figure 4.**
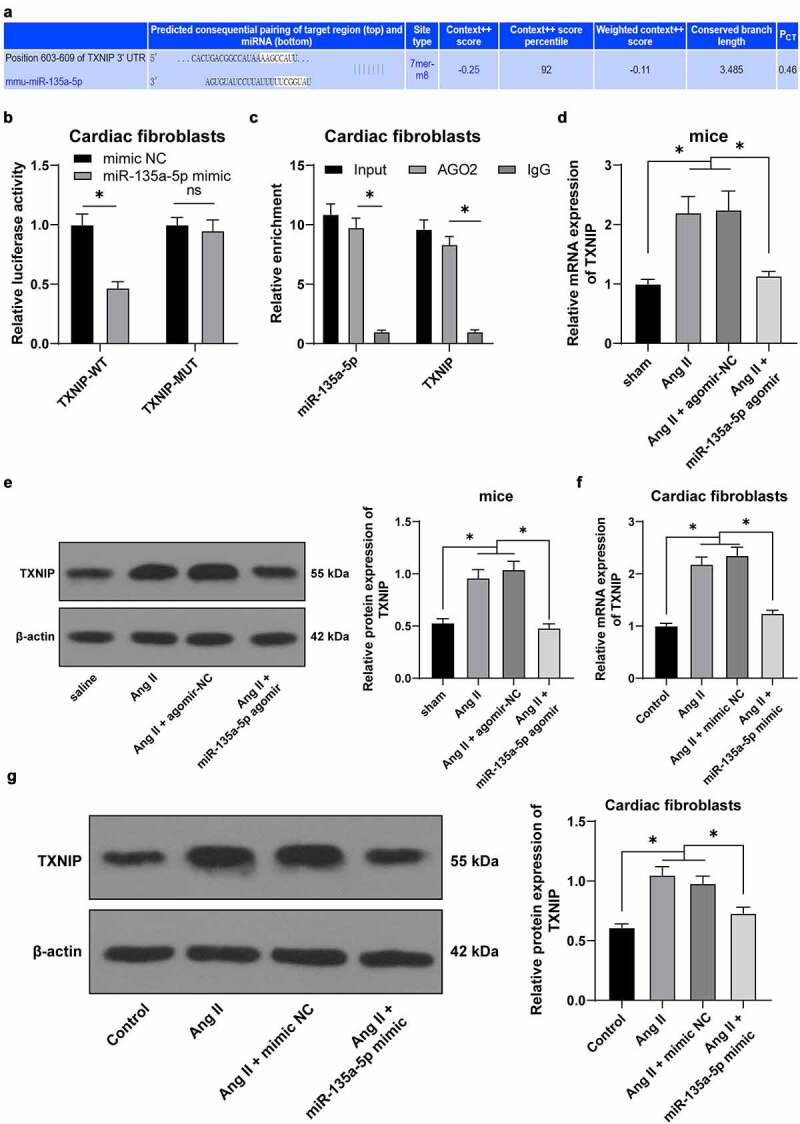
*miR-135a-5p* negatively regulated *TXNIP*. a: The targeting relationship of *miR-135a-5p* and *TXNIP* predicted via ENCORI: The Encyclopedia of RNA Interactomes. (sysu.edu.cn) website. b-c: The targeting relationship of *miR-135a-5p* and *TXNIP* testified via dual-luciferase reporter and RIP assays; d: *TXNIP* expression in cardiac tissues of hypertensive mice detected via RT-qPCR and Western blot analysis; f-g: *TXNIP* expression in mouse cardiac fibroblasts determined via RT-qPCR and Western blot analysis. Cell experiments were performed 3 times, and data were represented as mean ± SD. Data in figures A-B were analyzed using t test. Data in figures c and g were analyzed using one-way ANOVA. Data in figures a-b and d-g were analyzed using one-way ANOVA, and data in figure C were analyzed using followed by Tukey’s multiple comparison test. * P < 0.05.

To further explore the downstream mechanism of *miR-135a-5p* in hypertension-induced cardiac fibrosis, *TXNIP* was predicted to be a downstream target of *miR-135a-5p* through the ENCORI: The Encyclopedia of RNA Interactomes. (sysu.edu.cn) website (Figure 4(a)). Meanwhile, there is evidence to suggest that *TXNIP* knockdown can alleviate Ang II-mediated cardiac fibrosis and hypertrophy [26]. Besides, prior studies have shown that *TXNIP* can further bind to *NLRP3* to regulate inflammation [42]. Additionally, we speculated that *miR-135a-5p* inhibits *TXNIP* to affect the binding of *TXNIP* to *NLRP3*, thus participating in hypertension-induced cardiac fibrosis and inflammation. *miR-135a-5p* was found to bind to *TXNIP* by means of dual-luciferase reporter and RIP assay (*P* < 0.05, Figure 4(b,c)). Furthermore, *TXNIP* patterns in cardiac tissues of different treatment groups were detected *via* RT-qPCR and Western blotting. It was found that *TXNIP* was up-regulated in cardiac tissues of Ang II–induced hypertensive mice, while inhibited in response to *miR-135a-5p* agomir (*P* < 0.05, Figure 4(d,e)). Besides, cell experimentation revealed that *miR-135a-5p* mimic could markedly inhibit Ang II–induced *TXNIP* up-regulation (*P* < 0.05, Figure 4(f-g)). Collectively, the aforementioned findings indicated that *miR-135a-5p* could negatively regulate *TXNIP*.

### miR-135a-5p *over-expression reduced the binding of* TXNIP *and* NLRP3

**Figure 5.**
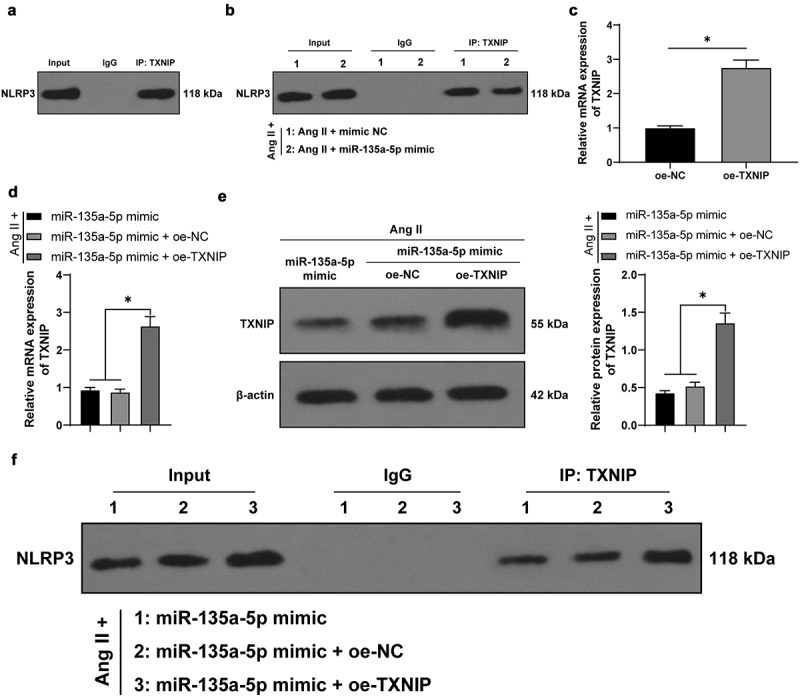
*miR-135a-5p* overexpression reduced the binding of *TXNIP* and *NLRP3*. a: Binding of *TXNIP* and *NLRP3* in mouse CFs detected via Co-IP assay; b: Binding of *TXNIP* and *NLRP3* upon transfection of *miR-135a-5p* mimic detected via Co-IP assay; c: *TXNIP* expression upon transfection of with oe-*TXNIP* determined via RT-qPCR; d: *TXNIP* expression detected upon transfection of *miR-135a-5p* mimic and oe-*TXNIP* via RT-qPCR and Western blot analysis; f: Interactive binding of *TXNIP* and *NLRP3* detected via Co-IP assay. Cell experiments were performed 3 times and data were represented as mean ± SD. Data in figure C were analyzed using t test. Data in figures D-E were analyzed using one-way ANOVA, followed by Tukey’s multiple comparison test. * P < 0.05.

Furthermore, we adopted a Co-IP assay and found that there is an interactive binding of *TXNIP* and *NLRP3* in mouse CFs (Figure 5(a)). Ang II-treated mouse CFs were transfected with mimic NC or *miR-135a-5p* mimic. The results of Co-IP revealed that the binding between *TXNIP* and *NLRP3* was reduced upon *miR-135a-5p* over-expression (Figure 5(b)). Meanwhile, *TXNIP* was found to be over-expressed in mouse CFs *via* oe-*TXNIP* plasmid (*P* < 0.05, Figure 5(c)). Ang II-treated mouse CFs were simultaneously transfected with *miR-135a-5p* mimic and oe-*TXNIP*, and subsequent experimentation showed that oe-*TXNIP* reversed the inhibitory role of *miR-135a-5p* mimic in *TXNIP* (*P* < 0.05, Figure 5(d,e)) and facilitated the binding of *TXNIP* and *NLRP3* (Figure 5(f)). Taken all, the aforementioned findings indicated that *TXNIP* and *NLRP3* could bind to each other, such that *miR-135a-5p* over-expression reduced the binding of *TXNIP* and *NLRP3*.

### miR-135a-5p *over-expression targeted* TXNIP *to suppress* NLRP3*-mediated inflammation and fibrosis of cardiac fibroblasts (CFs)*

**Figure 6.**
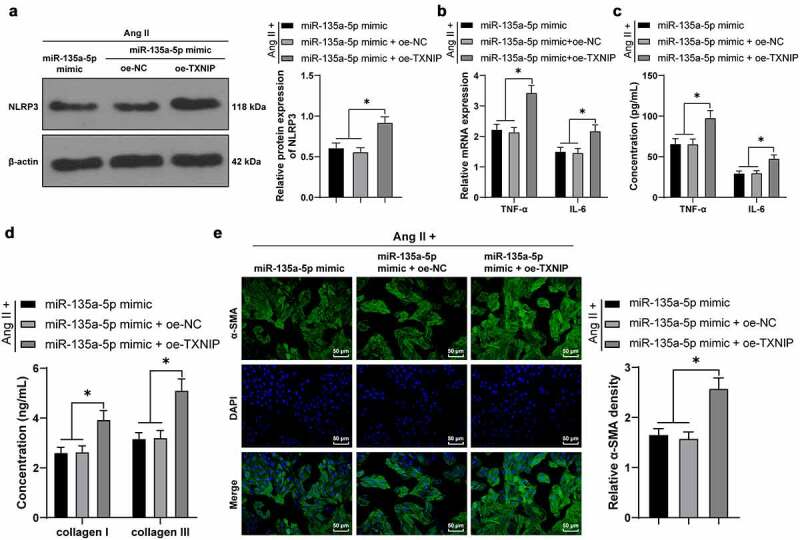
*miR-135a-5p* overexpression targeted *TXNIP* to suppress *NLRP3*-mediated inflammation and fibrosis of CFs. Ang II-treated mouse CFs were simultaneously transfected with *miR-135a-5p* mimic and oe-*TXNIP*. a: *NLRP3* expression detected via Western blot analysis; b-c: TNF-α and IL-6 concentrations measured via RT-qPCR and ELISA; d: COL-I and COL-III expressions detected via ELISA; e: a-SMA expression detected via immunofluorescence. Cell experiments were performed 3 times and data were represented as mean ± SD. Data in figures a and e were analyzed using one-way ANOVA and data in figures b-d were analyzed using two-way ANOVA, followed by Tukey’s multiple comparison test. * P < 0.05.

Lastly, we testified the crosstalk of *miR-135a-5p* and the *TXNIP*/*NLRP3* axis in CFs. Briefly, the Ang II-treated mouse CFs were simultaneously transfected with *miR-135a-5p* mimic and oe-*TXNIP*. Subsequent results of Western blotting, RT-qPCR, and ELISA showed that compared with the *miR-135a-5p* mimic + oe-NC group, *NLRP3*, TNF-α, and IL-6 expression levels were all significantly augmented in the *miR-135a-5p* mimic + oe-*TXNIP* group (*P* < 0.05, Figure 6(a-c)). In addition, ELISA also illustrated that compared with the *miR-135a-5p* mimic + oe-NC group, COL-I and COL-III expression levels were markedly elevated in the *miR-135a-5p* mimic + oe-*TXNIP* group (*P* < 0.05, Figure 6(d)). Moreover, immunofluorescence detection showed that compared with the *miR-135a-5p* mimic + oe-NC group, a-SMA expression levels were markedly up-regulated in the *miR-135a-5p* mimic + oe-*TXNIP* group (*P* < 0.05, Figure 6(e)). Altogether, the above-mentioned findings indicated that *miR-135a-5p* over-expression targeted *TXNIP* to suppress *NLRP3*-mediated inflammation and fibrosis of CFs.

## Discussion

Hypertension serves as a critical factor in the induction of cardiac remodeling, further manifested by inflammation and fibrosis of the myocardium [43,44]. Interestingly, the hard-done works of our peers have highlighted the involvement of miRNAs in the epigenetic regulation of hypertensive heart diseases [45]. In the current study, we set out to analyze the functional mechanism of *miR-135a-5p* in cardiac inflammation, and the obtained findings indicated that over-expression of *miR-135a-5p* inhibits *TXNIP* expression to reduce the binding of *TXNIP* and *NLRP3*, thereby alleviating cardiac inflammation and fibrosis.

Firstly, we established hypertensive mouse models by means of Ang II induction and uncovered that *miR-135a-5p* was markedly decreased in Ang II-treated mice. Similarly, prior studies have documented poor expressions of miR-135a upon isoproterenol-induced cardiac fibrosis, wherein miR-135a was indicated to play a therapeutic role in cardiac fibrosis treatment [22,23]. To further analyze the role of *miR-135a-5p* in hypertensive cardiac fibrosis, Ang II-treated mice were intramyocardially injected with *miR-135a-5p* agomir. Subsequent findings illustrated *miR-135a-5p* agomir brought about a reduction in blood pressure and improvements in LVEF and LVFS, which were suggestive of improved cardiac functions of hypertensive mice. Furthermore, histopathologic examination illustrated that *miR-135a-5p* agomir diminished Ang II–induced inflammatory infiltration and almost eliminated cardiac fibrosis. Meanwhile, there is a plethora of evidence to underscore the cardioprotective effects of *miR-135a-5p*. For instance, previous studies have shown that up-regulation of *miR-135a-5p* fights against hypoxia-induced cardiomyocyte injury [46], wehreas also protects cardiomyocytes from doxorubicin-induced oxidative stress and apoptosis [47]. Altogether, the aforementioned findings and evidence make it plausible to suggest that *miR-135a-5p* exerts cardioprotective functions and alleviates cardiac fibrosis of hypertensive mice.

Additionally, hypertension is known to contribute to macrophage polarization and inflammatory responses, further precipitating cardiac remodeling [43]. Meanwhile, existing evidence suggests that *NLRP3* plays a synergistic role with other pro-inflammatory cytokines, such as TNF-α and IL-6, thereby contributing to myocardial injury [48]. It is also noteworthy that activation of *NLRP3* inflammasome plays a critical role in the initiation of cardiac inflammation and fibrosis [41]. Herein, our experiments revealed that *NLRP3* was up-regulated by Ang II, while down-regulated in response to *miR-135a-5p* agomir. On a separate note, TNF-α and IL-6 are well-established inflammatory cytokines, which are further enriched in CFs upon activation of inflammation [49]. We came across an increase in TNF-α and IL-6 levels following Ang II treatment, whereas *miR-135a-5p* agomir exhibited the opposite trends, which underscores the anti-inflammatory phenotype of *miR-135a-5p* in hypertensive mice. On top of that, we isolated CFs from mice and treated them with Ang II, followed by transfection with *miR-135a-5p* mimic. It was observed that Ang II facilitated the expressions of *NLRP3*, TNF-α, and IL-6, while *miR-135a-5p* mimic reversed the aforementioned trends of inflammatory cytokines. Essentially, cardiac fibrosis is generally initiated by collagen deposition, and is further evidenced by alterations of COL-I, COL-III, and a-SMA [50]. We learned that Ang II elevated the expressions of COL-I, COL-III, and a-SMA, and unsurprisingly, *miR-135a-5p* mimic reversed these protein changes. In accordance with our findings, prior studies have illustrated that *miR-135a-5p* is druggable with schisandrin B to block the activation of *NLRP3* inflammasome, consequently mitigating airway inflammation [51]; and miR-135a is also capable of repressing inflammation signaling pathways, α-SMA expression, and augmented E-cadherin expression to ultimately relieve pulmonary fibrosis [52]. Altogether, the aforementioned *in vitro* and *in vivo* findings elucidated that *miR-135a-5p* attenuated cardiac inflammation and fibrosis in hypertensive mice and Ang II-treated CFs.

Furthermore, we explored the downstream mechanism of *miR-135a-5p*, and the initial results of online database prediction directed our focus to *TXNIP*. In addition, prior research has indicated that *miR-135a-5p* possesses the ability to repress *TXNIP* to retard the activation of the *NLRP3* inflammasome, in so doing attenuating ischemic brain injury [24]. On the other hand, *TXNIP* is capable of binding to *NLRP3* to induce nerve cell injury [53]. Accordingly, we validated the binding relationship between *miR-135a-5p* and *TXNIP* with the help of dual-luciferase reporter and RIP assays. Meanwhile, prior studies have identified that *TXNIP* is up-regulated in mice with diabetic cardiomyopathy, such that *TXNIP* knockdown averts the stimulating role of Ang II in cardiac fibrosis [26]. Herein, our findings illustrated that *TXNIP* was up-regulated by Ang II in cardiac tissues while undergoing down-regulation in response to *miR-135a-5p* mimic, which indicated that *miR-135a-5p* negatively regulated *TXNIP* to antagonize the effects of Ang II. Besides, the results of Co-IP assay revealed that *TXNIP* and *NLRP3* could indeed bind to each other, whereas *miR-135a-5p* over-expression inhibited the binding of *TXNIP* and *NLRP3*. Additionally, we simultaneously transfected Ang II-treated CFs with *miR-135a-5p* mimic and oe-*TXNIP*, and observed that *TXNIP* over-expression reversed the inhibitory role of *miR-135a-5p* mimic in *TXNIP* and facilitated the binding of *TXNIP* and *NLRP3*. Moreover, *TXNIP* over-expression facilitated the inflammation and fibrosis of CFs, as manifested by augmented expressions of *NLRP3*, TNF-α, IL-6, COL-I, COL-III, and a-SMA. What’s noteworthy is that the role of *TXNIP* in inflammation and fibrosis of CFs is consistent with its role in proximal tubule epithelial cells, hepatocytes, and lung fibroblasts [15,54,55]. Overall, our findings evidenced that *miR-135a-5p* inhibits *TXNIP* to suppress *NLRP3*-inflammasomme mediated inflammation and fibrosis of CFs. To the best of our knowledge, our study is the first-of-its-kind to shed a light on the mechanism of *miR-135a-5p* targeting *TXNIP* to restrict *NLRP3*-inflammasomme activation in CFs.

## Conclusion

In summary, findings uncovered in the current study highlighted the protective role of *miR-135a-5p* in *NLRP3*-mediated cardiac inflammation and fibrosis through the mechanism wherein *miR-135a-5p* inhibited *TXNIP* to reduce the binding of *TXNIP* and *NLRP3*. However, there are a few limitations to our study. For instance, we failed to detect *miR-135a-5p* agomir-mediated cytotoxicity and which type of cells it affects or investigate the regulatory role of abnormally low expressions of *miR-135a-5p* in hypertensive mice and the function of *miR-135a-5p* in hypertension-induced fibrosis of other organs. In addition, our study was limited in functional assessment and exploration of other signaling pathways that may be involved in hypertension-induced fibrosis. Besides, since results from mice may be different from humans, there is still a need to validate the transition of obtained findings into clinical application. Meanwhile, the NLRP3 inflammasome consists of Caspase1, ASC, IL-1b, etc., whereas the protein levels of Caspase1, ASC, and other proteins are implicated in specific inflammatory cell death modes, such as pyroptosis, which is a deeper regulatory mechanism and also the future direction of our study. In that light, we were unable to perform any detection of the expression patterns of Caspase1, ASC, and IL-1b in the current study. In the future, we will perform more functional assessment and interference of *miR-135a-5p* down-regulation, explore other signaling pathways that affect cardiac fibrosis, and evaluate the role of *miR-135a-5p* in hypertension-induced organ damage and further validate the role of *miR-135a-5p* in human-derived CFs.

## Supplementary Material

Supplemental Material

## Data Availability

The datasets used and analyzed during the current study are available from the corresponding author on reasonable request.

## References

[1] Aras MA, Psotka MA, De Marco T. Pulmonary hypertension due to left heart disease: an update. Curr Cardiol Rep. 2019;21:62. 31134443 10.1007/s11886-019-1149-1

[2] Kokubo Y, Matsumoto C. Hypertension is a risk factor for several types of heart disease: review of prospective studies. Adv Exp Med Biol. 2017;956:419–426. 27815926 10.1007/5584_2016_99

[3] Tichelbacker T, Dumitrescu D, Gerhardt F, et al. Pulmonary hypertension and valvular heart disease. Herz. 2019;44:491–501. 31312873 10.1007/s00059-019-4823-6

[4] Stacey RB, Hundley WG. Integrating measures of myocardial fibrosis in the transition from hypertensive heart disease to heart failure. Curr Hypertens Rep. 2021;23:22. 33881630 10.1007/s11906-021-01135-8

[5] Schelbert EB. Myocardial scar and fibrosis: the ultimate mediator of outcomes?. Heart Fail Clin. 2019;15:179–189. 30832810 10.1016/j.hfc.2018.12.009

[6] Espeland T, Lunde IG, and Ha B, et al. Myocardial fibrosis. Tidsskr Nor Laegeforen. 2018;138. doi:10.4045/tidsskr.17.1027. 30344312

[7] Shenasa M, Shenasa H. Hypertension, left ventricular hypertrophy, and sudden cardiac death. Int J Cardiol. 2017;237:60–63. 28285801 10.1016/j.ijcard.2017.03.002

[8] Sweeney M, Corden B, Cook SA. Targeting cardiac fibrosis in heart failure with preserved ejection fraction: mirage or miracle?. EMBO Mol Med. 2020;12:e10865. 32955172 10.15252/emmm.201910865 PMC7539225

[9] Shao BZ, Xu ZQ, Han BZ, et al. *NLRP3* inflammasome and its inhibitors: a review. Front Pharmacol. 2015;6:262. 26594174 10.3389/fphar.2015.00262 PMC4633676

[10] Wang Z, Zhang S, Xiao Y, et al. *NLRP3* inflammasome and inflammatory diseases. Oxid Med Cell Longev. 2020;2020:4063562. 32148650 10.1155/2020/4063562 PMC7049400

[11] De Miguel C, Pelegrin P, Baroja-Mazo A, et al. Emerging role of the inflammasome and pyroptosis in hypertension. Int J Mol Sci. 2021;22:1064. 33494430 10.3390/ijms22031064 PMC7865380

[12] Pinar AA, Scott TE, Huuskes BM, et al. Targeting the *NLRP3* inflammasome to treat cardiovascular fibrosis. Pharmacol Ther. 2020;209:107511. 32097669 10.1016/j.pharmthera.2020.107511

[13] Liang Q, Cai W, Zhao Y, et al. Lycorine ameliorates bleomycin-induced pulmonary fibrosis via inhibiting *NLRP3* inflammasome activation and pyroptosis. Pharmacol Res. 2020;158:104884. 32428667 10.1016/j.phrs.2020.104884

[14] Mridha AR, Wree A, Robertson AAB, et al. *NLRP3* inflammasome blockade reduces liver inflammation and fibrosis in experimental NASH in mice. J Hepatol. 2017;66:1037–1046. 28167322 10.1016/j.jhep.2017.01.022 PMC6536116

[15] Wu M, Han W, Song S, et al. *NLRP3* deficiency ameliorates renal inflammation and fibrosis in diabetic mice. Mol Cell Endocrinol. 2018;478:115–125. 30098377 10.1016/j.mce.2018.08.002

[16] Song W, Zhang T, Yang N, et al. Inhibition of micro RNA miR-122-5p prevents lipopolysaccharide-induced myocardial injury by inhibiting oxidative stress, inflammation and apoptosis via targeting GIT1. Bioengineered. 2021;12:1902–1915. 34002676 10.1080/21655979.2021.1926201 PMC8806731

[17] Pan J, Xu Z, Guo G, et al. Circ_nuclear factor I X (circNfix) attenuates pressure overload-induced cardiac hypertrophy via regulating miR-145-5p/ATF3 axis. Bioengineered. 2021;12:5373–5385. 34468254 10.1080/21655979.2021.1960462 PMC8806771

[18] Ding Y, Wang J, Lu J. miR-337-5p promotes the development of cardiac hypertrophy by targeting Ubiquilin-1 (UBQLN1). Bioengineered. 2021;12:6771–6781. 34515612 10.1080/21655979.2021.1964892 PMC8806775

[19] Wang S, Cheng Z, Chen X, et al. microRNA-135a protects against myocardial ischemia-reperfusion injury in rats by targeting protein tyrosine phosphatase 1B. J Cell Biochem. 2019;120:10421–10433. 30644128 10.1002/jcb.28327

[20] Feng H, Xie B, Zhang Z, et al. MiR-135a protects against myocardial injury by targeting TLR4. Chem Pharm Bull (Tokyo). 2021;69:529–536. 34078799 10.1248/cpb.c20-01003

[21] Chen C, Shen H, Huang Q, et al. The circular RNA CDR1as regulates the proliferation and apoptosis of human cardiomyocytes through the miR-135a/HMOX1 and miR-135b/HMOX1 axes. Genet Test Mol Biomarkers. 2020;24:537–548. 32762552 10.1089/gtmb.2020.0034

[22] Wei Y, Wu Y, Feng K, et al. Astragaloside IV inhibits cardiac fibrosis via miR-135a-TRPM7-TGF-beta/Smads pathway. J Ethnopharmacol. 2020;249:112404. 31739105 10.1016/j.jep.2019.112404

[23] Wu Y, Liu Y, Pan Y, et al. MicroRNA-135a inhibits cardiac fibrosis induced by isoproterenol via TRPM7 channel. Biomed Pharmacother. 2018;104:252–260. 29775892 10.1016/j.biopha.2018.04.157

[24] Liu Y, Li YP, Xiao LM, et al. Extracellular vesicles derived from M2 microglia reduce ischemic brain injury through microRNA-135a-5p/*TXNIP*/*NLRP3* axis. Lab Invest. 2021;101:837–850. 33875790 10.1038/s41374-021-00545-1

[25] Ferreira NE, Omae S, Pereira A, et al. Thioredoxin interacting protein genetic variation is associated with diabetes and hypertension in the Brazilian general population. Atherosclerosis. 2012;221:131–136. 22236479 10.1016/j.atherosclerosis.2011.12.009

[26] Rao Y, Chen J, Guo Y, et al. Rivaroxaban ameliorates angiotensin II-induced cardiac remodeling by attenuating *TXNIP*/Trx2 interaction in KKAy mice. Thromb Res. 2020;193:45–52. 32521334 10.1016/j.thromres.2020.05.030

[27] Guide for the care and use of laboratory animals. 8th. Washington (DC); 2011. doi:10.17226/12910.

[28] Watanabe K, Narumi T, Watanabe T, et al. The association between microRNA-21 and hypertension-induced cardiac remodeling. PLoS One. 2020;15:e0226053. 32040481 10.1371/journal.pone.0226053 PMC7010249

[29] Li Y, Zhang H, Li Z, et al. microRNA-130a-5p suppresses myocardial ischemia reperfusion injury by downregulating the HMGB2/NF-kappaB axis. BMC Cardiovasc Disord. 2021;21:121. 33658008 10.1186/s12872-020-01742-4 PMC7931544

[30] Lyu L, Chen J, Wang W, et al. Scoparone alleviates Ang II-induced pathological myocardial hypertrophy in mice by inhibiting oxidative stress. J Cell Mol Med. 2021;25:3136–3148. 33560596 10.1111/jcmm.16304 PMC7957216

[31] Meng J, Qin Y, Chen J, et al. Treatment of hypertensive heart disease by targeting Smad3 signaling in mice. Mol Ther Methods Clin Dev. 2020;18:791–802. 32953930 10.1016/j.omtm.2020.08.003 PMC7475647

[32] Meijles DN, Cull JJ, Markou T, et al. Redox regulation of cardiac ASK1 (Apoptosis Signal-Regulating Kinase 1) controls p38-MAPK (Mitogen-Activated Protein Kinase) and orchestrates cardiac remodeling to hypertension. Hypertension. 2020;76:1208–1218. 32903101 10.1161/HYPERTENSIONAHA.119.14556 PMC7480944

[33] Li T, Chen Y, Li Y, et al. FAM134B-mediated endoplasmic reticulum autophagy protects against sepsis myocardial injury in mice. Aging (Albany NY). 2021;13:13535–13547. 33819192 10.18632/aging.202786 PMC8202901

[34] Huang XR, Chung AC, Yang F, et al. Smad3 mediates cardiac inflammation and fibrosis in angiotensin II-induced hypertensive cardiac remodeling. Hypertension. 2010;55:1165–1171. 20231525 10.1161/HYPERTENSIONAHA.109.147611

[35] Wang F, Trial J, Diwan A, et al. Regulation of cardiac fibroblast cellular function by leukemia inhibitory factor. J Mol Cell Cardiol. 2002;34:1309–1316. 12392991 10.1006/jmcc.2002.2059

[36] Zhang W, Wang Q, Feng Y, et al. MicroRNA-26a protects the heart against hypertension-induced myocardial fibrosis. J Am Heart Assoc. 2020;9:e017970. 32865120 10.1161/JAHA.120.017970 PMC7726969

[37] Zhao M, Yang Y, Li J, et al. Silencing of OIP5-AS1 protects endothelial cells from ox-LDL-triggered injury by regulating KLF5 expression via sponging miR-135a-5p. Front Cardiovasc Med. 2021;8:596506. 33778018 10.3389/fcvm.2021.596506 PMC7994260

[38] Livak KJ, Schmittgen TD. Analysis of relative gene expression data using real-time quantitative PCR and the 2(-Delta Delta C(T)) method. Methods. 2001;25:402–408. 11846609 10.1006/meth.2001.1262

[39] Li JH, Liu S, Zhou H, et al. starBase v2.0: decoding miRNA-ceRNA, miRNA-ncRNA and protein-RNA interaction networks from large-scale CLIP-Seq data. Nucleic Acids Res. 2014;42:D92–97. 24297251 10.1093/nar/gkt1248 PMC3964941

[40] Li Z, Guo Z, Lan R, et al. The poly(ADP-ribosyl)ation of BRD4 mediated by PARP1 promoted pathological cardiac hypertrophy. Acta Pharm Sin B. 2021;11:1286–1299. 34094834 10.1016/j.apsb.2020.12.012 PMC8148063

[41] Gan W, Ren J, Li T, et al. The SGK1 inhibitor EMD638683, prevents Angiotensin II-induced cardiac inflammation and fibrosis by blocking *NLRP3* inflammasome activation. Biochim Biophys Acta Mol Basis Dis. 2018;1864:1–10. 28986310 10.1016/j.bbadis.2017.10.001

[42] Chen D, Dixon BJ, Doycheva DM, et al. IRE1alpha inhibition decreased TXNIP/NLRP3 inflammasome activation through miR-17-5p after neonatal hypoxic-ischemic brain injury in rats. J Neuroinflammation. 2018;15:32. 29394934 10.1186/s12974-018-1077-9 PMC5797348

[43] Mouton AJ, Li X, Hall ME, et al. Obesity, hypertension, and cardiac dysfunction: novel roles of immunometabolism in macrophage activation and inflammation. Circ Res. 2020;126:789–806. 32163341 10.1161/CIRCRESAHA.119.312321 PMC7255054

[44] Nadruz W. Myocardial remodeling in hypertension. J Hum Hypertens. 2015;29:1–6. 24804791 10.1038/jhh.2014.36

[45] Verjans R, Peters T, Beaumont FJ, et al. MicroRNA-221/222 family counteracts myocardial fibrosis in pressure overload-induced heart failure. Hypertension. 2018;71:280–288. 29255073 10.1161/HYPERTENSIONAHA.117.10094

[46] Xu JJ, Zheng WH, Wang J, et al. Long non-coding RNA plasmacytoma variant translocation 1 linked to hypoxia-induced cardiomyocyte injury of H9c2 cells by targeting *miR-135a-5p*/forkhead box O1 axis. Chin Med J (Engl). 2020;133:2953–2962. 33093283 10.1097/CM9.0000000000001147 PMC7752684

[47] Wang X, Cheng Z, Xu J, et al. Circular RNA Arhgap12 modulates doxorubicin-induced cardiotoxicity by sponging *miR-135a-5p*. Life Sci. 2021;265:118788. 33245966 10.1016/j.lfs.2020.118788

[48] Yu Y, Hu LL, Liu L, et al. Hsp22 ameliorates lipopolysaccharide-induced myocardial injury by inhibiting inflammation, oxidative stress, and apoptosis. Bioengineered. 2021;12:12544–12554. 34839787 10.1080/21655979.2021.2010315 PMC8810130

[49] Hanna A, Frangogiannis NG. Inflammatory cytokines and chemokines as therapeutic targets in heart failure. Cardiovasc Drugs Ther. 2020;34:849–863. 32902739 10.1007/s10557-020-07071-0 PMC7479403

[50] Jin D, Han F. FOXF1 ameliorates angiotensin II-induced cardiac fibrosis in cardiac fibroblasts through inhibiting the TGF-beta1/Smad3 signaling pathway. J Recept Signal Transduct Res. 2020;40:493–500. 32496870 10.1080/10799893.2020.1772299

[51] Chen X, Xiao Z, and Jiang Z, et al. Schisandrin B attenuates airway inflammation and airway remodeling in asthma by inhibiting *NLRP3* inflammasome activation and reducing pyroptosis. Inflammation. 2021;44(6): 2217–2231. 34143347 10.1007/s10753-021-01494-z

[52] Xie B, Lu C, Chen C, et al. miR-135a alleviates silica-induced pulmonary fibrosis by targeting NF-kappaB/inflammatory signaling pathway. Mediators Inflamm. 2020;2020:1231243. 32617074 10.1155/2020/1231243 PMC7317310

[53] Liu W, Zhang G, Sun B, et al. Activation of NLR family, domain of pyrin containing 3 inflammasome by nitrous oxide through thioredoxin-interacting protein to induce nerve cell injury. Bioengineered. 2021;12:4768–4779. 34348577 10.1080/21655979.2021.1954741 PMC8806838

[54] Han -Y-Y, Gu X, Yang C-Y, et al. Protective effect of dimethyl itaconate against fibroblast–myofibroblast differentiation during pulmonary fibrosis by inhibiting *TXNIP*. J Cell Physiol. 2021;236(11):7734–7744. 34061990 10.1002/jcp.30456

[55] Park HS, Song JW, Park JH, et al. *TXNIP*/VDUP1 attenuates steatohepatitis via autophagy and fatty acid oxidation. Autophagy. 2021;17:2549–2564. 33190588 10.1080/15548627.2020.1834711 PMC8496541

