# Supplementary figures and images for: microRNA-135a-5p regulates NOD-like receptor family pyrin domain containing 3 inflammasome-mediated hypertensive cardiac inflammation and fibrosis via thioredoxin-interacting protein
Source: Bioengineered. 2022 Feb 11;13(3):4658–73. doi: 10.1080/21655979.2021.2024956 (PMC8973706; doi:10.1080/21655979.2021.2024956)

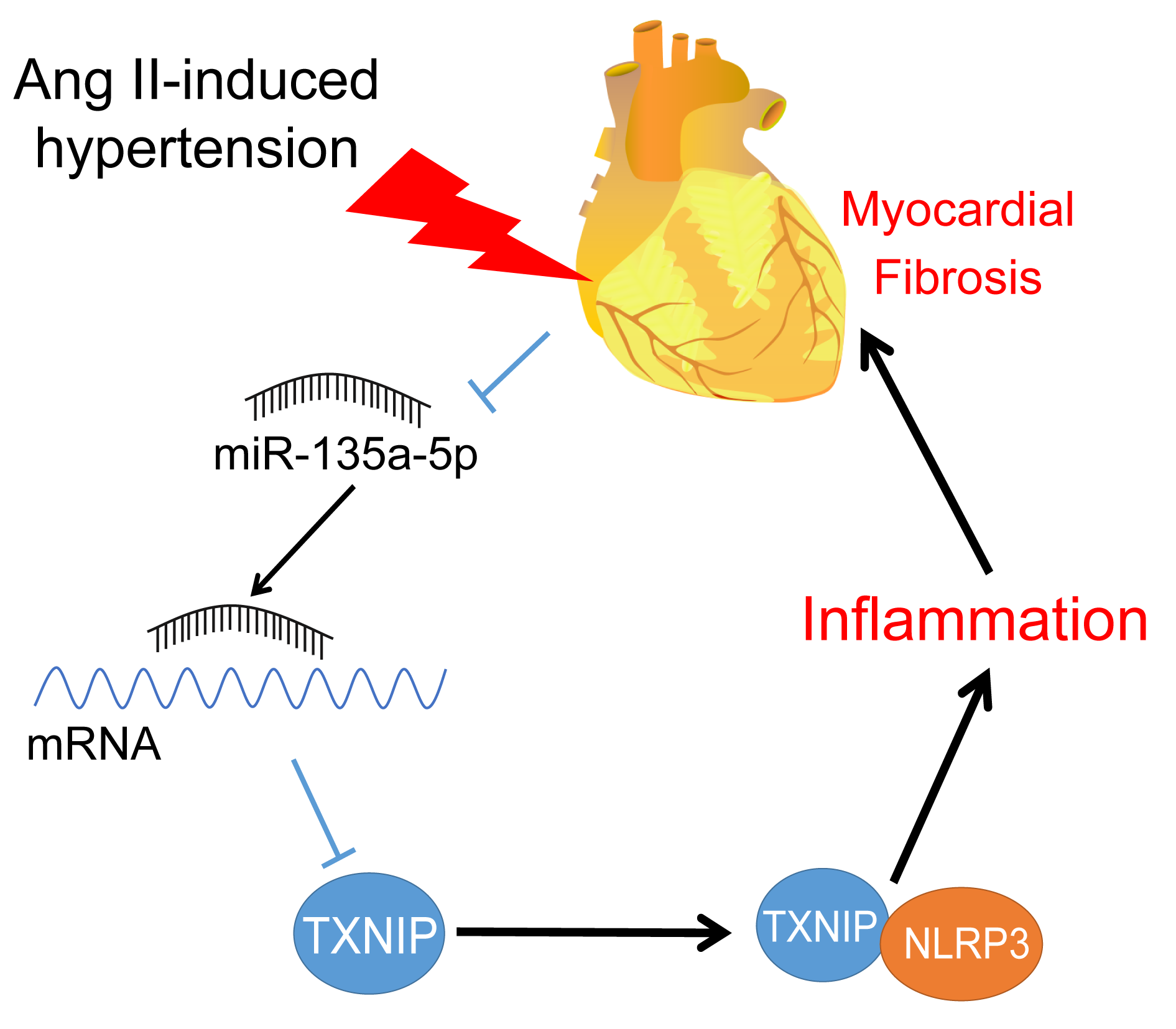

Supplement: Supplemental Material [file KBIE_A_2024956_SM7816.zip › supplementary/revised Graphical Abstract.tiff]
